# The epistemic injustice of borderline personality disorder

**DOI:** 10.1192/bji.2024.16

**Published:** 2024-11

**Authors:** Jay Watts

**Affiliations:** Honorary Senior Lecturer, Centre for Mental Health Research, City University, University of London, London, UK. Email: clinic@jaywatts.co.uk

**Keywords:** Personality disorders, borderline personality disorder, epistemic injustice, testimonial injustice, patients and service users

## Abstract

Borderline personality disorder (BPD) has been a controversial diagnosis for over 40 years. It was to be removed from the latest version of the ICD, only to be reintroduced as a trait qualifier as a result of last-minute lobbying. Retaining BPD as a de facto diagnosis keeps us stuck at a deadlock that undermines the voices of patients who have persistently told us this label adds ‘insult to injury’. Miranda Fricker's concept of epistemic injustice helps illuminate how this affects subjectivity and speech, hermeneutically sealing patients in ways of thinking that are not evidence-based, resulting in testimonial smothering (altering or withholding one's narratives) and testimonial quieting (dismissing a speaker's capacity to provide worthy testimony) that prevent more affirmative explanations.

Borderline personality disorder (BPD) remains among the most passionately disputed diagnoses in psychiatry.^1^ Its nosological origins can be traced back to the transformative era of DSM-III in 1980, when its inclusion acted as a concession to the psychoanalytic fraternity, sparking widespread dissatisfaction among task force members.^2^ This tendency to yield to the status quo has been a consistent theme throughout the subsequent evolution of personality pathology. The taskforces for both ICD-10 and DSM-IV leaned towards a shift in dimensional representations, only to be abruptly pulled back on the cusp of ratification. The recently unveiled ICD-11, despite its pivot to a dimensional framework, chose to retain BPD as a trait qualifier at the last moment, succumbing to the pressure of political lobbying.^3^

The result is a seemingly endless debate that pleases no one. Researchers find themselves burdened with a de facto diagnosis that collapses the new statistical model.^1,3^ Clinicians grapple with a diagnosis so heterogeneous and overlapping with many other conditions, such as autism, attention-deficit hyperactivity disorder (ADHD), bipolar disorder and complex post-traumatic stress disorder (PTSD), that it jeopardises the credibility of diagnostic systems.^1,3^ For patients, any validation or explanation the diagnosis purports to offer is transient, considering the relentless controversy shadowing the label.^1^

Above all, this impasse silences the decades-long outcry from survivor and patient groups.^1,4^ These groups have continuously told us that the BPD construct confirms their worst fears about themselves, enabling iatrogenic care that retraumatises them.^1,4^ The concept of epistemic justice, introduced by philosopher Miranda Fricker, can help us understand the encaging nature of the BPD construct and break the current deadlock.

## Understanding epistemic injustice

Fricker's seminal work *Epistemic Injustice: Power and the Ethics of Knowing* introduces two main forms of epistemic injustice.^5^ The first is testimonial injustice, where a speaker's credibility is devalued owing to harmful bias. The second is hermeneutical injustice, which arises when a collective lack of interpretive resources hinders the understanding of certain social experiences of specific groups. These concepts have become invaluable tools for reassessing power dynamics in psychiatry,^6^ including within the context of BPD,^7^ with Fricker's notion of ‘testimonial sensibility’ becoming operationally useful^8^ in considering what environments enable listening and hearing to become possible.

Expanding on Fricker's ideas, Kristie Dotson's work on testimonial silencing^9^ sheds light on how credibility of testimonials can be undermined through self-censorship and premature dismissal. She identified the concepts of testimonial smothering, where speakers alter or withhold narratives to avoid misunderstandings, and testimonial quieting, where listeners prematurely dismiss a speaker's credibility. Now, let us explore how these concepts play out in the context of BPD.

## Epistemic injustice in BPD – Laura's story

Meet Laura, a 25-year-old woman who grew up in what she described as a rough neighbourhood, enduring sexual abuse at church, where her mother had sent her in the hope of providing stability. The diagnosis of BPD hit Laura like a sharp blow, as she had always attributed her emotional struggles to the traumatic experiences she endured during her formative years. Being told she had a personality disorder felt like an insult rather than a helpful diagnosis,^4,7^ instantly bringing back painful memories: a shopkeeper muttering under his breath that he did not want ‘her kind’ in his shop and her mother's drunken remark that Laura should never have been born.

As Laura went through her dialectical behaviour therapy (DBT) sessions, she was overwhelmed by intense feelings of shame, anger and deep humiliation. Every attempt to open up about her traumatic experiences was met with redirection to skills training, leaving Laura feeling that she had done something wrong and her pain was being dismissed. The hurtful stereotypes about patients with BPD being labelled as attention-seeking, manipulative and difficult had already haunted her,^4,7,10^ and when she bravely challenged her diagnosis, her voice seemed to hit a wall of indifference. In the psychoeducational component of DBT, she felt silenced and unseen. Her profound frustration reached a tipping point, and one day she could not contain it any longer. Laura left the group room abruptly, accidentally overturning a chair in her desperation to escape the emotional confinement. However, instead of understanding her distress, her keyworker deemed her disruptive and began planning her discharge, adding to her sense of isolation and vulnerability.

## The implications of testimonial injustice

We can see how problematic this type of scenario is using Fricker's framework. Psychiatric diagnoses can both inflate and deflate testimonial credibility, depending on the specific diagnosis and the context. Diagnoses such as obsessive–compulsive disorder (OCD) or depression are more likely to inflate testimonial credibility as they legitimise suffering, providing a tool to bat away micro-aggressions such as ‘I get sad too’ or ‘Yeah, I always go back to check the oven’. Conversely, diagnoses such as schizophrenia and personality disorders are far more likely to deflate testimonial credibility. Schizophrenia does this by attacking the speaker's rationality, through the notion of lack of insight. BPD does so by not only individualising problems that have been relationally unseen or unregistered, such as trauma or undiagnosed autism,^1^ but locating them in problems with one's very being rather than a condition, illness or divergence one has. This is especially epistemically harmful as it attacks the person's character, slurring their very moral essence and framing them as what Dotson terms ‘a bad affective investment’.^9^

**Fig. 1:**
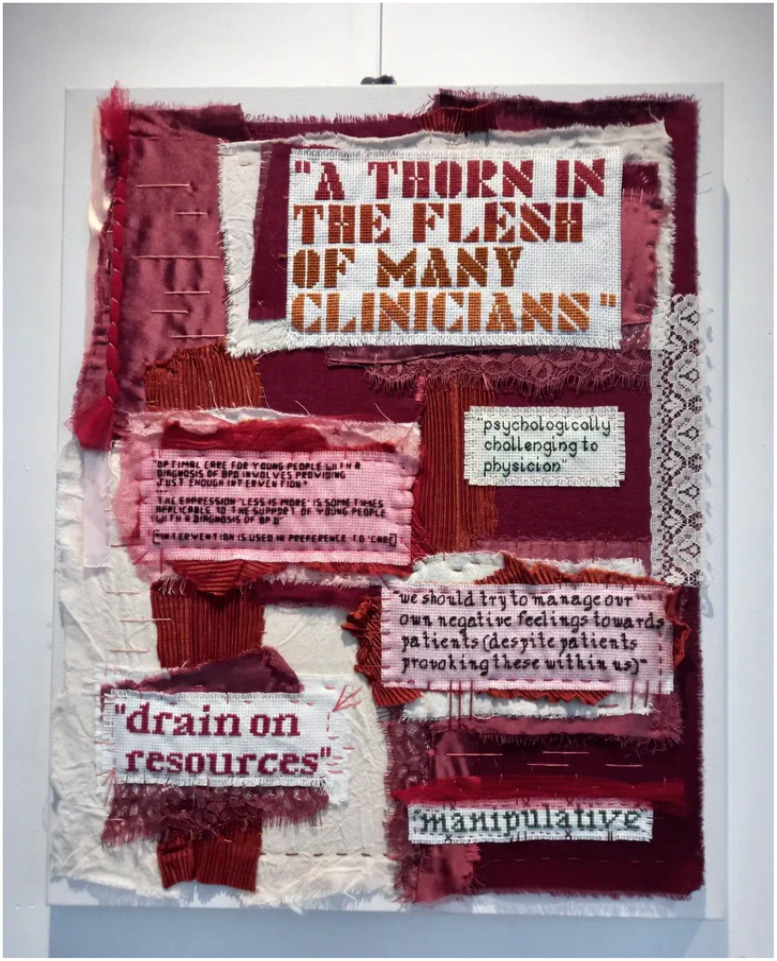
*Appliqué* by Bekah Harris for the ‘BPD: Beyond the Label’ exhibition, hosted by 42nd Street, Manchester, May–June 2022. Permission granted by the artist to publish photo of their exhibit.

This discourse enables clinicians to accept and perpetuate the harmful ‘heartsink’ stereotype associated with BPD^7,10^ without unsettling their ideas of themselves as helpers. Patients often face belittling, contradictory responses, including avoidance, withdrawal of warmth, rejection and reluctance to provide care^1,4^ owing to the lingering idea that BPD is not a genuine mental illness, but rather portrays patients as ‘attention-seeking’, ‘manipulative’ and ‘difficult’ (Fig. 1).^10^ Consequently, this can lead to maltreatment and dismissal of patients through DARVO (deny, attack, and reverse victim and offender) tactics.^11^ Psychoanalytic concepts such as ‘splitting’, which can deny patients access to clinicians they feel safer with, and ‘projective identification’, which enables the expression of feelings of hatred and disgust that would be unacceptable in other contexts,^2,7^ are intimately entwined with the idea of BPD, serving as a ‘personality disorder shield’.^12^

The insidious nature of the BPD construct goes beyond mere perception, as it extends its stifling grasp into the very core of one's self-perception and lived experience.^4,7^ Through testimonial smothering and quietening attempts to speak, as seen in Laura's case when she tried to share her trauma, the construct effectively silences and invalidates individual perspectives. It unyieldingly shapes the narrative of a person's life, forcibly weaving together past and present into a constricted storyline that reflects the notion of an inherent character flaw. Laura's experiences in the shop and her mother's remark become evidence of this basic character flaw, positioning them as reflective of her problem rather than being partly constitutive of it. These hurtful moments, etched into her memory, are woven into a narrative that reinforces her self-doubts and vulnerabilities.

**Fig. 2:**
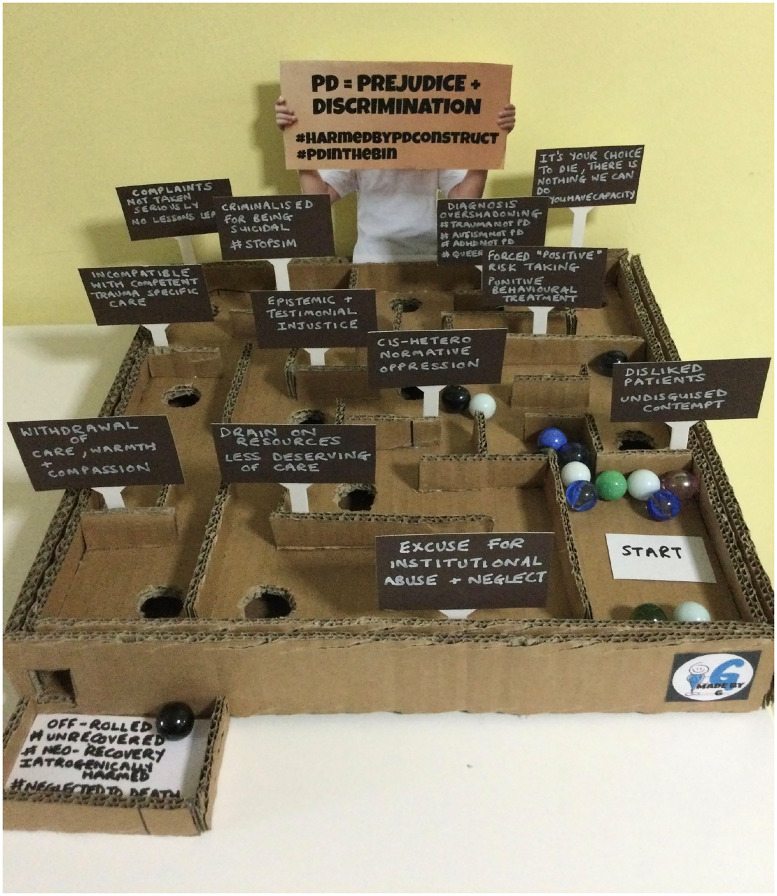
Exhibit by Jee for the art exhibition *A Sophisticated Insult*, hosted by No Format Gallery, London, July 2023. Permission granted by the artist to publish photo of their exhibit.

This establishment of a predetermined narrative is both merciless and baseless, exaggerating claims and imposing a sense of inevitable doom. Despite evidence that 85% of individuals with the BPD label achieve recovery at 10 years,^3^ the label provokes more negative clinician ratings of problems and prognosis than a more neutral behavioural description does (Fig. 2).^1^ Yet, we must be cautious of the idea that recovery is inevitable here, not least as this may reduce access to services. The outcome data suggest that ongoing functional problems often persist – just not ones that are symptomatically centred in the BPD construct.^1^ Once again, the construct's centring on symptoms clinicians find challenging renders an invisibility, masking lingering symptoms that have never been hermeneutically registered as such because they have not particularly troubled clinicians, even though they are profoundly damaging to the course of a life. This oversight perpetuates the cycle of epistemic injustice, hermetically sealing off from view many of the real struggles of those diagnosed.

The ripple effects of this testimonial injustice can reverberate through various aspects of life. In family courts, it is used to undermine childcare rights. In healthcare settings, it is associated with ‘hysteria’, minimising legitimate complaints of physical health concerns and overshadowing genuine medical diagnoses. In romantic relationships, the construct invalidates and dismisses female emotional responses and fears, heightening the risk of revictimisation and perpetuating cycles of harm.

## Breaking the deadlock

This epistemic harm is exacerbated by BPD's status as a closed system, serving as a hermeneutic seal, meaning almost any behaviour can be explained within its logic, including refusing to accept the diagnosis.^7^ Given that BPD is such a heterogeneous category,^1,3^ it is almost impossible not to identify with or be identified by it. Who amongst us has not had problems managing our emotions or our relationships, or been unsure about who we are as a teenager? What woman, especially, has not got caught in the spider's web of paradoxical demands placed on femininity – adapt to whoever you are with but be stable, be pleasing but not seductive – without becoming overwhelmed, exhausted and self-destructive at times?

We could laugh at BPD's crystallisation of such old-fashioned ideas, but we cannot ignore that these expectations are deeply ingrained in our cultures, leading to a double-entry bookkeeping effect where two separate explanatory accounts are held at the same time. Even survivors who question the diagnosis with enquiries such as ‘How exactly do you expect an abuse victim to behave?’ find themselves wrestling with an internalised perpetrator who carries not only the legacy of early abusive figures but is emboldened by personality disorder's character slur that, in feeling so familiar, reinforces the label's validity.^7^ This leads many to perceive the BPD label as a form of medicalised victim-blaming,^1,4^ whether the internalised perpetrator has been a sexual predator or, to give but one other example, a neurotypical world that has persistently placed all the problems in the patient.

Efforts to remove the diagnosis face opposition from professionals who argue that the BPD label is liked by some patients, who have often been told that intensive therapy is dependent on it. This argument involves two misconceptions. The first presumes patients possess the autonomy to disassociate from a BPD diagnosis, thus equating them with those who find the label helpful. This overlooks the self-reinforcing nature of the BPD construct; even the rejection of the label can be interpreted as symptomatic of the disorder, making it impossible to choose whether to identify with the diagnosis or not. Second, the argument suggests that the benefits of diagnosis – providing explanation, enabling access to treatment and facilitating a sense of community – are not achievable through other means.

These misconceptions result in a hermeneutic injustice. They give rise to testimonial smothering and silence patients harmed by the diagnosis, stunting both scientific and humanitarian progress. It is in addressing this epistemic injustice that we need to muster our hermeneutic resources. Clear communication can alleviate fears of what might be lost in removing the BPD label, particularly by highlighting the scientific and ethical problems with the construct^1,3^ and by emphasising the availability of more affirmative alternative explanations^1,3,4^ to maintain diagnostic rights for those who require them.

More validating alternative diagnoses, such as autism, bipolar disorder, premenstrual dysphoric disorder and complex PTSD, not only better explain the heterogeneity but are free from character assassination, with its devastating deflation of testimonial credibility, although these must be available alongside non-medicalised pathways for trauma survivors should they wish them.^4^ This stance is not a dereliction of the evidence-base, considering the transdiagnostic nature of all recommended treatments that target common features of serious mental distress.^1^ Rather, it is a recognition that the BPD construct often hinders access to help and ripples into unintended areas of life^1,4^ and that, more than 40 years after BPD was first introduced in DSM-III as a patchwork solution that pleased no one,^2^ we can do better. Getting rid of the BPD label can be framed as a win-win for all. So, isn't it about time that we move beyond the straitjacket of the BPD label and devote our energies to building sensitive, non-judgemental, non-stigmatising frameworks for delivering care?

## Data Availability

Data availability is not applicable to this article as no new data were created or analysed in this study.
